# Broad‐Spectrum Antibacterial Activity of Postbiotic From *Lacticaseibacillus paracasei* BGP1 Against Multidrug‐Resistant Skin Wound Pathogens

**DOI:** 10.1002/mbo3.70137

**Published:** 2025-12-08

**Authors:** Farideh Mohammadhosseinzadeh, Ehsan Arefian, Mouj Khaleghi, Hoda Keshmiri Neghab, Nasim Kashef

**Affiliations:** ^1^ Department of Microbiology, School of Biology, College of Science University of Tehran Tehran Iran; ^2^ Department of Biology, Faculty of Science Shahid Bahonar University of Kerman Kerman Iran; ^3^ Department of Medical Laser, Medical Laser Research Center Yara Institute, ACECR Tehran Iran

**Keywords:** antibacterial activity, antibiotic resistance, *Lacticaseibacillus paracasei* BGP1, organic acids, postbiotic, skin wound infection

## Abstract

The increasing problem of antibiotic resistance indicates the need for alternative therapeutic strategies for skin wound infections. While probiotics exhibit potential for developing such alternatives, the majority of antimicrobial studies focus on live cells or lysates and oral delivery. Notably, in dermatology, formulating products with live strains poses technical challenges due to stability issues in water‐based systems. Postbiotics, substances made from probiotics, offer a promising, stable, and safe alternative. This study addresses the gap by evaluating the antibacterial potential of cell‐free supernatants (CFSs) from six probiotic strains, with a specific focus on *Lacticaseibacillus paracasei* BGP1, against clinically relevant skin pathogens. CFSs were screened in vitro using agar well diffusion and broth microdilution assays against *Staphylococcus aureus* ATCC 25923, methicillin‐resistant *S. aureus* (MRSA; UTMC 1442), *Pseudomonas aeruginosa* ATCC 27853, and *P. aeruginosa* PAO1. GC‐MS analysis was used to identify bioactive compounds in the most promising CFS. Among the tested strains, BGP1 demonstrated both consistent inhibitory (MIC: 6.25 mg/mL) and bactericidal (MBC: 12.5 mg/mL) effects. GC‐MS analysis identified palmitic acid (33.24%) and stearic acid (46.45%) as dominant bioactive compounds. These findings provide novel evidence that postbiotic metabolites from *L. paracasei* BGP1 represent a promising, broad‐spectrum, stable, and nonliving candidate to conventional therapies for antibiotic‐resistant skin wound infections. Further in vivo research is needed to evaluate their therapeutic potential and formulation in clinical settings.

## Graphical abstract

Postbiotic metabolites from *Lacticaseibacillus paracasei* BGP1 showed strong inhibitory and bactericidal activity against *Pseudomonas aeruginosa, Pseudomonas aeruginosa* PAO1, *Staphylococcus aureus*, and MRSA. Palmitic and stearic acids were identified as dominant bioactive compounds, supporting BGP1 postbiotics as stable and promising candidates for treating antibiotic‐resistant skin wound infections.

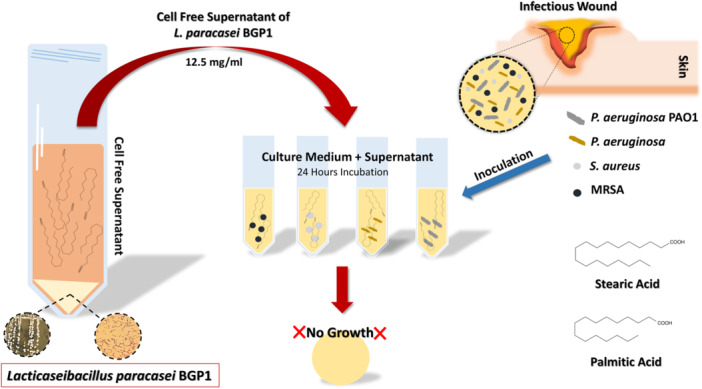

## 1 Introduction

The skin, as the body's largest organ, functions as an essential barrier in the innate immune system (Byrd et al. 2018). When the skin is compromised, it becomes susceptible to the development of an acute or chronic wound. Chronic wounds, such as diabetic foot ulcers and pressure sores, exhibit delayed healing and are frequently worsened by bacterial infections (Tsiouris et al. 2017; Ferro et al. 2019; Fijan et al. 2019). The nutrient‐rich skin wound environment facilitates bacterial colonization and biofilm formation, leading to sustained inflammation, hindering healing, and promoting the emergence of chronic wounds (Tsiouris et al. 2017; Ferro et al. 2019). *Staphylococcus aureus* (SA) (gram‐positive cocci) and *Pseudomonas aeruginosa* (PA) (rod‐shaped, gram‐negative) are among the most prevalent and troublesome pathogens. Both demonstrate a strong biofilm‐forming capacity and increasing resistance to antibiotics, including MRSA and multidrug‐resistant *P. aeruginosa* (Serra et al. 2015; Al‐Mebairik et al. 2016; Pirlar 2020; Tuon et al. 2022).

*S. aureus* is generally identified in the superficial layer of wounds, whereas *P. aeruginosa* is isolated from the deeper areas of the wound bed (Serra et al. 2015). Wounds infected with *P. aeruginosa* are characterized by larger lesion areas and a delayed healing process (Serra et al. 2015; Ferro et al. 2019). This bacterium has shown both intrinsic and acquired resistance to antibiotics, complicating its eradication (Tuon et al. 2022). Likewise, *P. aeruginosa* is a biofilm‐forming bacterium in the wound bed. Biofilm production strengthens the virulence and pathogenesis of bacterium (Serra et al. 2015). *S. aureus* is associated with numerous skin disorders and is characterized by many virulence characteristics, including biofilm development. It mainly colonizes in chronic and non‐healing wounds as a resistant biofilm against antimicrobial treatments (Melo et al. 2016). In addition, the remarkable fact is that the synergistic and competitive interaction between *S. aureus* and other opportunistic pathogenic species, particularly *P. aeruginosa*, exacerbates virulence, enhances antibiotic resistance, and prolongs the healing of infectious skin wounds (Farahpour et al. 2017; Algburi et al. 2021). Effective treatment of *staphylococcal* infections can be problematic due to the emergence and spread of drug‐resistant strains like MRSA (Al‐Mebairik et al. 2016).

The increasing prevalence of antibiotic‐resistant pathogens, especially *S. aureus* and *P. aeruginosa*, poses big challenges in wound management by delaying healing, increasing the risk of chronic wound development, and complicating treatment protocols. A report from the United States indicates that skin infections, such as burns, surgical site infections, and untreated diabetic foot ulcers, affect approximately two million individuals annually, leading to 200,000 fatalities and incurring over $18 billion in direct medical expenses (Kadam 2019). These infections not only contribute substantially to global morbidity and mortality but also place a considerable economic burden on healthcare systems through both direct treatment expenses and indirect costs, such as lost productivity and early retirement. Furthermore, the prolonged nature of such infections can severely impair patients' mental health and overall quality of life (Ferro et al. 2019; Olsson et al. 2019).

Antibiotics have traditionally served as the principal therapeutic agents in the management of chronic wound infections, administered both topically and systemically. However, systemic dosing is not recommended in specific instances, such as diabetic wounds with biofilm, due to variable tissue concentrations and insufficient supporting evidence. Conversely, topical application encounters limitations, including inadequate drug penetration, the existence of biofilms, tissue inflammation, and the challenge of targeting multiple pathogens simultaneously. Although a diverse array of antibiotics exists, their efficacy is frequently undermined by factors such as insufficient tissue penetration and the development of resistance. Extended or excessive usage, particularly in localized infections or systemic involvement, not only accelerates the emergence of antibiotic‐resistant strains but is also frequently associated with serious side consequences (Jager et al. 2019; Smith et al. 2020; Mohtashami et al. 2021). Thus, there is a vital need for accessible and cost‐effective therapeutic interventions with minimal side effects to combat microbial colonization and facilitate effective skin regeneration. Given these challenges, there is an increasing interest in alternative, biologically based strategies that provide both efficacy and safety. Alternative approaches, including using beneficial microbes for pathogen control, are increasingly being recognized, and probiotics are emerging as promising candidates. Probiotics are defined as live microorganisms that, when administered in adequate amounts, confer health benefits beyond basic nutrition (Tazehabadi et al. 2021; Mbarga Manga Joseph 2022). Balancing the gut microbiota, enhancing epithelial barrier integrity, and modulating immune and inflammatory responses are just some of their documented properties (Tsiouris and Tsiouri 2017; Fijan et al. 2019; Mohtashami et al. 2021). The probiotics' antimicrobial mechanisms encompass competitive exclusion of pathogens, production of antimicrobial substances, and modulation of the host immune system (Tsiouris and Tsiouri 2017; Cizeikiene and Jagelaviciute 2021; Lee 2022; Mbarga Manga Joseph 2022). Besides live probiotic cells, cell‐free probiotic derivatives or bioactive metabolites, such as organic acids, bacteriocins, and hydrogen peroxide, known as postbiotics, collectively proved to have notable antimicrobial effects (Busarcevic and Dalgalarrondo 2012; Ogura 2018; Pirlar 2020; McLoughlin et al. 2022).

Notwithstanding the widespread recognition of postbiotics' antimicrobial potential, their clinical application remains a topic of debate. Most research is still focused on the effects of live cells or lysates, mainly through oral delivery routes, leaving topical applications relatively underexplored. This gap is particularly significant in dermatology, where incorporating live strains into topical formulations poses technical challenges due to their instability in water‐based systems like creams and lotions. Considering this issue, it seems that paying attention to postbiotics as safer, more stable, and cost‐effective alternatives to probiotics in controlling infection in dermatology could be promising and more efficient. In topical skin treatments, postbiotics offer clear advantages over probiotics: they are more resilient to heat and moisture, less likely to provoke infections or inflammatory responses, do not transmit potential antibiotic resistance to the host, and are easier and cheaper to formulate, store, and evaluate in the pharmaceutical and food industries (Aguilar‐Toalá et al. 2018).

Despite the promising potential of postbiotics for wound care, particularly in treating antibiotic‐resistant and biofilm‐associated chronic infections, current knowledge regarding their composition, mechanisms of action, optimal dosing, and therapeutic efficacy remains limited. Furthermore, for industrial‐scale production, understanding the optimal timing for postbiotic harvest, aligned with the dynamic growth phases of probiotic cells, is essential to maximize bioactivity and cost‐efficiency. Addressing these research gaps is critical to advancing the development of effective, antibiotic‐free therapies for chronic wound management. Given this, the present work investigated the potential of probiotic‐derived cell‐free supernatants (CFSs), or postbiotics, as safe and cost‐effective alternatives to probiotics for controlling skin wound infections caused by antibiotic‐resistant strains of *SA* and *PA*.

The probiotic strains used for this study, including LR LRE 02 (DSM 2387), *Lactobacillus acidophilus* (LA) LA3, *Lacticaseibacillus casei* (LCP) LC03 (DSM 27537), *Lacticaseibacillus paracasei* (LPC) BGP1, *Lacticaseibacillus rhamnosus* LR 06 (DSM 21981), and *Bifidobacterium animalis* BB12, were selected based on their established safety profiles and prior use in food products and oral applications, thus ensuring a solid foundation for future translational research. Additionally, they are readily available from commercial sources in Iran, making them feasible for future development and potential commercialization.

The aforementioned strains above are among the most common and safe probiotics used in the food industry and have been predominantly investigated in the context of food preservation and gastrointestinal health (Margiotta et al. 2021; Chamignon et al. 2023; Roozbahani et al. 2024; Yolmeh et al. 2025). Considering this, we hypothesized that they may serve as promising and potentially more reliable sources for inclusion in topical medicinal formulations. Nonetheless, probiotics' effects are strain‐specific and not universally applicable (Senok et al. 2005). Therefore, there is a critical need to understand the characteristics and production of postbiotics, metabolic profile, potential for topical application in infection control, and optimal effective dosage in dermatology.

## 2 Materials and Methods

### 2.1 Bacterial Strains and Culture Conditions

Probiotic strains: Six probiotic strains, *LR* LRE 02 (DSM 2387), LA LA3, *LCP* LC03 (DSM 27537), LPC BGP 1, *Lacticaseibacillus rhamnosus* (LRM) LR 06 (DSM 21981), and *Bifidobacterium animalis* BB12, were originally manufactured by Sacco S.r.l. (Italy), and Probiotical S.p.A. (Novara, Italy) and were commercially obtained from Pourateb Pharmaceutical Company (Tehran, Iran) and stored at −20°C. The strain was activated by inoculating the strains into liquid MRS broth (Ibresco, Iran) and incubating them under static and anaerobic conditions at 37°C for 24–48 h.

Pathogen strains: *SA* ATCC 25923 was obtained from the microbial stock collection of the Medical Bacteriology Laboratory (Microbiology Department, Faculty of Biology, University of Tehran, Tehran, Iran). *PA* ATCC 27853 was supplied by the Bu‐Ali Sina Hospital Stock Collection (Tehran, Iran). Methicillin‐resistant SA (MRSA; UTMC 1442), a clinical isolate from a chronic wound, was also used. PA PAO1 was kindly donated by Professor Michael R. Hamblin from the Wellman Center for Photomedicine, Harvard Medical School, USA. Cells were activated by culturing in Mueller‐Hinton (MH) broth (Ibresco, Iran) for 24 h at 37°C under aerobic conditions.

The antibiotic resistance profiles of the cultured strains were subsequently evaluated.

The names of the strains in the text are abbreviated as follows:

*Lacticaseibacillus casei* (LCP), *Limosilactobacillus reuteri* (LR), *Lactobacillus acidophilus* (LA), *Lacticaseibacillus paracasei* (LPC), *Lacticaseibacillus rhamnosus* (LRM), *Staphylococcus aureus* (SA), *Pseudomonas aeruginosa* (PA), PA PAO1 (PAO1), and methicillin‐resistant SA (MRSA).

### 2.2 Antibiotic Susceptibility Assay of Bacteria

Antibiotics were selected to cover a wide range of mechanisms of action, and they offer an in‐depth look at the resistance profile of both probiotic and pathogenic strains. Pathogenic and probiotic strains were screened for antibiotic susceptibility using the Kirby‐Bauer disk diffusion susceptibility test (Bauer 1996). For probiotic strains, susceptibility to 10 broad‐spectrum antibiotics (tetracycline, chloramphenicol, streptomycin, gentamicin, ciprofloxacin, imipenem, oxacillin, penicillin, trimethoprim‐sulfamethoxazole, and vancomycin) (Oxoid, UK) was evaluated. These antibiotics were selected because they target different bacterial cellular processes (e.g., protein synthesis, cell wall synthesis, nucleic acid synthesis, and folate metabolism). This comprehensive selection helps to identify probiotic strains and resistance traits that can be transferred to both the host and pathogen.

For pathogenic strains, susceptibility to 15 specific antibiotics (vancomycin, clindamycin, penicillin, oxacillin, piperacillin, doxycycline, ceftazidime, colistin) (Oxoid, UK) and general antibiotics (tetracycline, gentamycin, ciprofloxacin, trimethoprim‐sulfamethoxazole, chloramphenicol, levofloxacin, imipenem) (Oxoid, UK) was tested. Specific types are clinically relevant for the treatment of infections, and the broad‐spectrum types were used to compare resistance levels across classes.

Pathogenic and probiotic cultures were adjusted to a turbidity equivalent to a 0.5 McFarland standard and inoculated onto Mueller‐Hinton (MH) agar (Ibresco, Iran) and MRS agar plates (Ibresco, Iran), respectively. The plates were incubated at 37°C for 24 h, aerobically for pathogens and anaerobically for probiotics. The diameter of the inhibition zone was measured using a digital caliper and interpreted according to the Clinical and Laboratory Standards Institute (CLSI) guidelines, 2015 (CLSI 2015).

### 2.3 Probiotic Growth Curve and pH Measurements

The growth curve helps understand the physiological and metabolic functions of bacteria. The bacterial growth curves were determined using spectrophotometry (UV‐Vis spectrophotometer, Hyperion, Germany) by measuring the optical density (OD) at 600 nm, from 0 to 48 h. Simultaneously, pH variations were examined by a pH meter (Mettler Toledo, Switzerland). Analysis of the growth curve along with pH changes facilitates to determination of the optimal time for the production of antibacterial metabolites, especially organic acids, which act as an indicator of fermentation in lactic acid bacteria. These bacteria ferment carbohydrates, resulting in the production of organic acids and a subsequent reduction in pH. Monitoring pH changes during the growth phase helps in the identification of the peak period of production of these metabolites, which is crucial for increasing their antimicrobial effectiveness and comprehending the metabolic processes that contribute to the antibacterial activity of lactic acid bacteria (Hor and Liong 2014; Ishikawa et al. 2021).

The generation time (*G*) of the probiotics in the exponential growth phase was calculated using the following formula (Equation 1):

G=t/(3.3log(b/B)).

*G* = Generation time (min), *t* = time per generation (min), *b* = Optical density (OD) at the end of the time interval, and *B* = Optical density (OD) at the beginning of the time interval.

### 2.4 Probiotic CFS Preparation

To prepare CFS, the method described by Qadi, Rossoni, and Algburi was employed with some modifications (Rossoni et al. 2018; Qadi et al. 2023; Algburi et al. 2024). The probiotic strains were cultivated in MRS broth and incubated under static and anaerobic conditions for 48 h. After incubation, the bacterial cultures (with a turbidity of 1 McFarland) were centrifuged at 3000 × g for 10 min at 4°C to separate the bacterial cell bodies. The resulting supernatant was then filtered through a 0.22 µm cellulose acetate membrane filter (BIOFILL, USA) to ensure complete removal of bacterial cells. Finally, the sterile CFS was lyophilized via a freeze dryer (CHRIST, Germany) and stored at −20°C until further use. Lyophilization preserves sample content more effectively.

The sterility of the lyophilized CFS was checked by diluting it in MH broth and culturing it on MH agar before each test.

### 2.5 Evaluation of Antibacterial Potential

The production of metabolites with antibacterial activity in probiotic CFSs was evaluated using the agar well diffusion method, which is widely employed as an initial antimicrobial identification assay for screening potential antimicrobial agents (Sarmurzina et al. 2017; Al‐Dulaimi et al. 2021). CFSs were prepared from probiotic cultures at different time points (8, 16, 24, 32, 40, and 48 h) during the growth curve, as described in Section 2.3.

To assess antibacterial activity, bacterial suspensions of the indicator pathogens (PA and SA), with a turbidity of 0.5 McFarland, were spread evenly on the surface of MH agar (prepared with 0.7% agar). Thereafter, 100 µL of each CFS sample was added to the punched wells in the agar plates. A control containing MRS culture medium was considered to establish baseline conditions (Algburi et al. 2020).

The antibacterial activity of the 48‐h CFSs was further examined after neutralization and catalase treatment. Neutralization was performed by adjusting the pH of the CFS to 7.0 using 0.1 M NaOH. For catalase treatment, the CFS samples were incubated with 1 mg/mL catalase at 37°C for 30 min. Plates were incubated at 37°C for 24 h, aerobically. The diameters of the inhibition zones were measured with a digital caliper.

### 2.6 Determination of Minimum Inhibitory Concentration (MIC) and Minimum Bactericidal Concentration (MBC) of Lyophilized CFS

The prepared lyophilized CFS (Section 2.4) was quantitatively tested for effective antibacterial activity. The MIC and MBC were determined through the broth microdilution method in 96‐well microtiter plates (Weinstein and Lewis 2020; Algburi et al. 2024). Lyophilized CFS was reconstituted to the original volume with sterile MH broth before serial dilution. An eight‐fold serial dilution of reconstituted CFS (0.78–100 mg/mL) was prepared. Then, 0.5 McFarland overnight cultures of pathogenic bacteria were added to the wells. The plates were incubated at 37°C for 24 h. The MIC was determined as the lowest concentration of lyophilized CFS in the well that was clear and without visible bacterial growth. To ensure accurate MIC determination and minimize error, the optical density (OD) of the samples was measured at 600 nm by a UV‐Vis spectrophotometer (Hyperion, Germany). To determine MBC, 10 µL aliquots from each well showing no visible growth, including MIC and higher concentrations, were subcultured onto MH agar plates and incubated at 37°C for 24 h. Wells without bacterial growth were considered the MBC, defined as the lowest concentration of CFS that completely killed bacterial cells.

The antimicrobial tests were repeated with catalase‐treated and neutralized lyophilized CFS using a 0.1 M NaOH solution. All tests were performed in triplicate.

### 2.7 Evaluation of the Durability of the Inhibitory Effect of Lyophilized CFS

The durability of the bactericidal and inhibitory effects of lyophilized CFS was assessed over 72 h. Briefly, the previously determined MIC of each lyophilized CFS (Section 2.6) was added to the wells of a 96‐well microtiter plate containing MH broth and a bacterial pathogen suspension adjusted to 0.5 McFarland. Plates were incubated at 37°C under aerobic conditions, and bacterial growth (turbidity) was visually checked every 24 h for 72 h. The MBC of each lyophilized CFS, determined as described in Section 2.6, was applied to confirm the bactericidal effect. All experiments were performed in triplicate, with incubation conditions kept constant throughout.

### 2.8 Chemical Analysis of CFS by GC‐MS

To identify the antibacterial components produced by the probiotic strains, the ethyl acetate extract of the probiotic supernatant was analyzed via gas chromatography‐mass spectrometry (GC‐MS). GC‐MS analysis of the extracts was performed using a 7890 A GC‐System, Agilent Technologies, USA, gas chromatography coupled with a 5975 C VL MSD mass spectrometer system with a triple‐axis detector. An Rtx‐5 MS capillary column (length 30 m, inner diameter 0.25 mm, and film thickness 25 µm) with helium as a carrier gas (99.999%, a flow rate of 1 mL/min), and an ionization energy of 70 electron volts was used for separation. The oven temperature in the initial stage was 50°C for 1 min, followed by a temperature increase at a rate of 5°C/min to 270°C, then remained constant for 10 min. The injection chamber temperature was maintained at 250°C. The National Institute of Standards and Technology (NIST) version 2.1 was used as the library source.

### 2.9 Quantification of Protein Content in CFS via Bradford Assay

The Bradford colorimetric assay was employed to determine the protein content of the CFSs. This method quantifies proteins by measuring the absorbance of Coomassie Brilliant Blue G‐250 (Sigma, USA) at 595 nm, which correlates with protein concentration.

For this assay, 30 mg of lyophilized CFS powder was dissolved in 1 mL of sterile deionized water. Then, 40 µL of the resulting solution was mixed with 180 µL of Bradford reagent in each well. After a short incubation period, the absorbance of the samples was measured at 595 nm. The protein content was determined by comparing the absorbance of the sample with a standard curve generated with bovine serum albumin (BSA).

The BSA standard curve exhibited a linear response in the range of 0–3 mg/mL, with a coefficient of determination (*r*²) of 0.9987, indicating a high degree of linearity. All measurements were performed in triplicate.

### 2.10 Statistical Analysis

MIC and MBC values were statistically analyzed using two‐way ANOVA analysis of variance, followed by Tukey's honest significant difference post hoc test. Statistical significance was set at 5% (*p* ≤ 0.05).

## 3 Results

### 3.1 Antibiotic Susceptibility Assay of Probiotic and Pathogen Strains

Antibiotic susceptibility testing of the probiotic strains was performed to ensure their identity and provide preliminary characterization of the strains acquired from the commercial source. As shown in Table 1, all tested probiotic strains were resistant to vancomycin, oxacillin, and trimethoprim‐sulfamethoxazole but susceptible to tetracycline, chloramphenicol, and penicillin. The results indicate that some probiotic strains exhibit inherent resistance to specific antibiotics, such as vancomycin and oxacillin. Conversely, their sensitivity to broad‐spectrum antibiotics, including tetracycline, supports their safety profile.

**Table 1:** Antibiotic susceptibility of the probiotic strains.

Antibiotic	Probiotic strains				
	LPC	LCP	LA	LRM	LR
Vancomycin (V)	R	R	R	R	R
Gentamycin (GM)	R	R	R	R	I
Tetracycline (TE)	S	S	S	S	S
Ciprofloxacin (CP)	I	I	R	R	R
Trimethoprim‐sulfamethoxazole (SXT)	R	R	R	R	R
Streptomycin (S)	S	R	S	S	S
Imipenem (IPM)	S	S	S	I	S
Penicillin (P)	S	S	S	S	S
Chloramphenicol (C)	S	S	S	S	S
Oxacillin (OX)	R	R	R	R	R

Abbreviations: I, intermediate; LA, lactobacillus acidophilus LA3; LCP, *Lacticaseibacillus* casei LC03; LPC, *Lacticaseibacillus* paracasei BGP 1; LR, *Limosilactobacillus* reuteri LRE 02; LRM, *Lacticaseibacillus* rhamnosus LR 06; R, resistant, S, sensitive.

Table 2 presents the antibiotic susceptibility profiles of the pathogenic strains. All tested pathogenic strains were susceptible to gentamicin. Except for SA, all pathogenic strains were resistant to trimethoprim‐sulfamethoxazole. The gram‐negative pathogens (PA and PAO1) showed resistance to chloramphenicol and tetracycline, whereas the gram‐positive pathogens (SA and MRSA) were susceptible to these antibiotics. MRSA was resistant to ciprofloxacin and levofloxacin.

**Table 2:** Antibiotic susceptibility of the pathogenic strains.

Antibiotic	Pathogenic strains			
	*P. aeruginosa* ATCC27853	PAO1	*S. aureus* ATCC25923	MRSA
Vancomycin^a^ (V)	Nt	Nt	S	R
Gentamycin (GM)	S	S	S	S
Tetracycline (TE)	R	R	S	S
Ciprofloxacin (CP)	S	S	S	R
Trimethoprim‐sulfamethoxazole (SXT)	R	R	S	R
Clindamycin^a^ (CC)	Nt	Nt	S	S
Imipenem^b^ (IPM)	S	S	Nt	Nt
Penicillin^a^ (P)	Nt	Nt	R	R
Chloramphenicol (C)	R	R	S	S
Oxacillin^a^ (OX)	Nt	Nt	S	R
Piperacillin^b^ (PIP)	S	S	Nt	Nt
Levofloxacin (LOM)	S	I	S	R
Doxycycline^a^ (D)	Nt	Nt	S	S
Ceftazidime^b^ (CAZ)	S	R	Nt	Nt
Colistin^b^ (CL)	S	R	Nt	Nt

Abbreviations: I, intermediate; MRSA, methicillin‐resistant *Staphylococcus aureus*; Nt, not tested; PAO1, *Pseudomonas aeruginosa* PAO1; R, resistant; S, sensitive.

^a^ Specific antibiotics for gram‐positive pathogens.

^b^ Specific antibiotics for gram‐negative pathogens.

Among the specific antibiotics tested, MRSA exhibited resistance to both vancomycin and oxacillin, whereas SA was susceptible to them (Table 2). In contrast to PA, which was susceptible to colistin and ceftazidime, PAO1 was resistant to both antibiotics.

Understanding the antibiotic resistance patterns of pathogenic strains is essential for effective infection control. The results revealed distinct differences in the antibiotic susceptibility of gram‐positive and gram‐negative bacteria to broad‐spectrum agents, such as levofloxacin and chloramphenicol. The ineffectiveness of antibiotic treatments against drug‐resistant strains was evident from the data presented in Table 2. MRSA exhibited notable resistance to ciprofloxacin and levofloxacin, two commonly used fluoroquinolone antibiotics. Resistance was also observed against vancomycin and oxacillin, which are often regarded as last‐line therapies for severe MRSA infections. Similarly, PA PAO1 demonstrated resistance to colistin and ceftazidime, which are among the key antibiotics used to treat infections caused by this pathogen.

Among the antibiotics tested, gentamicin demonstrated strong antibacterial activity against the pathogenic strains, while exhibiting no inhibitory effect on the selected probiotic strains, except for LR. This selective activity is noteworthy. This selective activity highlights gentamicin as a favorable therapeutic option in situations where it is important to control pathogenic bacteria without disrupting beneficial probiotic populations.

### 3.2 Probiotic Growth and pH Measurements

**Figure 1:**
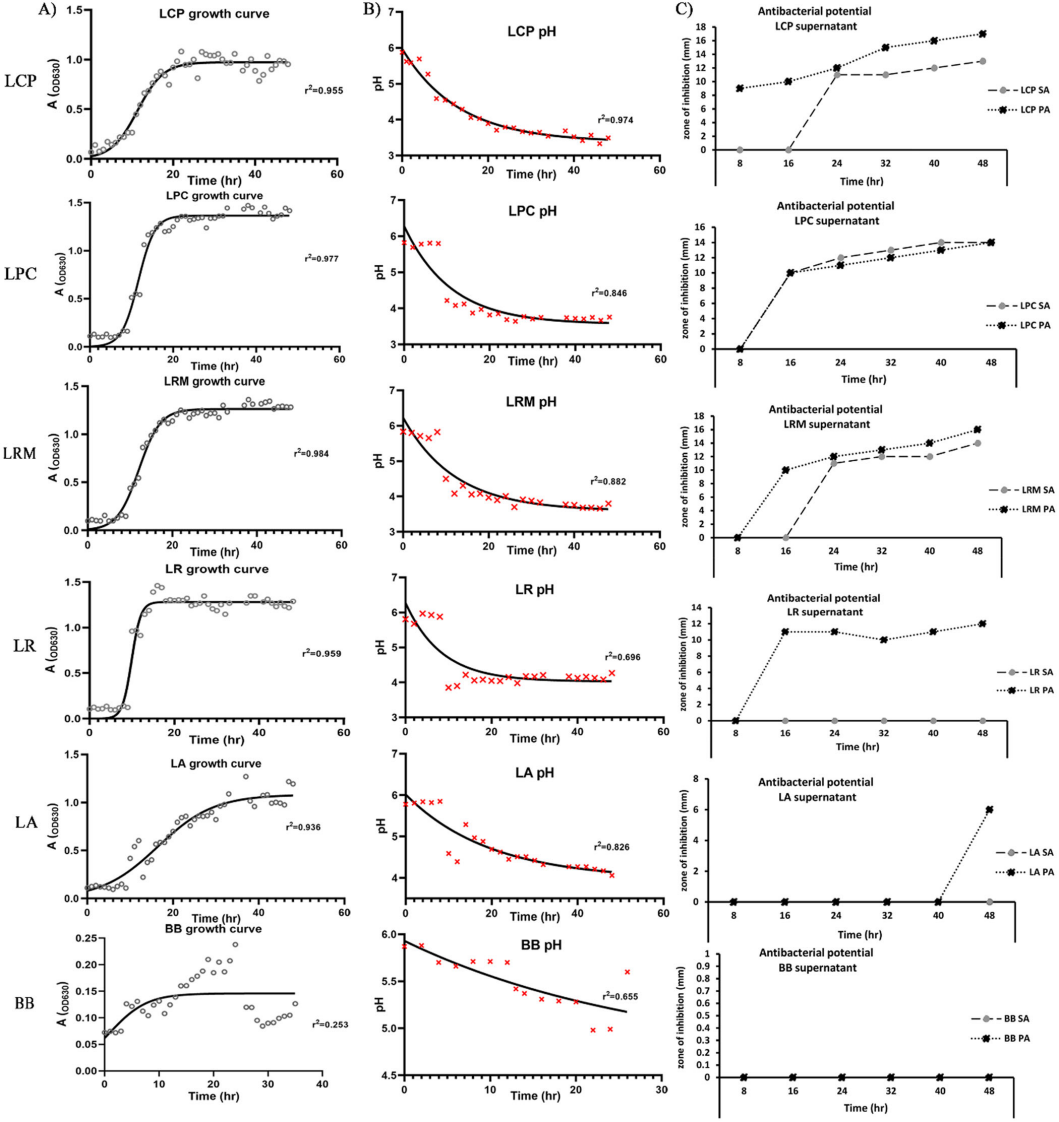
Growth dynamics and antibacterial activity of probiotic strains. Growth curves of probiotic strains *LCP, LPC, LRM, LR, LA*, and *BB* were measured by optical density at 600 nm (OD₆₀₀) over 48 h in MRS broth at 37°C. (A) and corresponding pH changes during incubation (B), Antibacterial activity of cell‐free supernatants collected at 8, 16, 24, 32, 40, and 48 h against *Staphylococcus aureus* (ATCC 25923) and *Pseudomonas aeruginosa* (ATCC 27853), showing time‐dependent inhibition patterns (C).

To assess the potential of postbiotics derived from selected probiotic strains as alternative antibacterial agents, their growth dynamics and pH changes were initially examined as indicators of metabolic activity and metabolite production. The growth curve and pH values shown in Figure 1 helped determine the optimal time for antibacterial metabolite production by probiotic strains LCP, LRM, LPC, LR, LA, and BB.

Based on Figure 1a, strains LRM, LR, LPC, and LA entered the logarithmic (exponential) phase at hour 9 of the incubation period. LCP and BB entered the logarithmic phase at hours 6 and 3, respectively. The onset of stationary phase varied among the strains. LRM, LPC, and LCP entered the stationary phase at hour 20. LA had a longer exponential phase, entering the stationary phase at hour 23. The logarithmic phase of LR lasted for 9 h. BB entered the stationary phase at hour 27 of the incubation period.

The growth and proliferation patterns of LRM, LR, LPC, and LA strains were similar, with LCP exhibiting a faster entry into the exponential phase. BB displayed a distinct growth pattern with a shorter growth period. The generation time of probiotic strains, calculated from the growth curve data, ranged from 20 to 64 min. Specifically, the generation times for LCP, LRM, LR, LPC, LA, and BB were 41.5, 38.2, 20.2, 36, 30.9, and 64.4 min, respectively.

The pH variations (Figure 1b) indicated the production of organic acids as end metabolites of lactic acid bacteria glucose fermentation (Hor and Liong 2014). A clear pH drop in each strain corresponded to the onset of the exponential phase; however, the extent of pH reduction varied. The maximum pH decrease was observed during the stationary phase and continued into the mid‐stationary phase. LCP reached the lowest pH values, ranging from 3.3 to 3.5. The pH values for LRM, LR, and LPC ranged from 3.6 to 4.1. LA exhibited a pH range of 4.0–4.5. In contrast, BB showed a different pattern. Whereas the logarithmic phase began at hour 3, the pH drop initiated at hour 12, reaching a pH of approximately 4.4.

This data elucidates the temporal dynamics of bacterial metabolite production and optimizes their yield from selected probiotic strains. Furthermore, it highlighted interstrain variability in growth kinetics and metabolite elaboration. Growth curves (Figure 1a) delineate the stationary phase, a period frequently associated with the maximal accumulation of secondary metabolites, including antibacterial compounds. Precise determination of each strain's entry into this phase facilitates accurate harvesting and maximizes product yield. Crucially, if metabolite production is phase‐specific, temporal characterization of that phase is essential. For instance, LR exhibited the shortest generation time and the highest growth rate, rendering it a potentially advantageous strain for industrial and large‐scale production. Collectively, these data provide a foundational framework for developing efficient and scalable processes for bacterial metabolite production, including antibacterial compounds, and empower researchers to select optimal probiotic strains based on metabolite profiles and production efficiency.

### 3.3 Antibacterial Potential Assay

Figure 1c presents the antibacterial activity of CFSs against SA and PA, as determined by the agar well diffusion method. As shown in Figure 1c, the supernatants of LRM, LR, LCP, and LPC exhibited antibacterial activity against both PA and SA. Inhibition zones were observed in wells containing supernatants from cultures incubated for 16, 24, 32, 40, and 48 h. The diameter of the inhibition zones generally increased with the incubation time of the bacterial cultures. The highest inhibition zone against PA (17 mm) and (16 mm) was observed with the 48‐h supernatant of LCP and LRM, respectively. The 48‐h supernatants of LPC and LRM exhibited the largest inhibition zones against SA), each measuring 14 mm. The supernatants LA and BB did not exhibit detectable antibacterial activity against either pathogen (Figure 1c).

The antibacterial activity of the 48‐h supernatants was further examined after neutralization (to pH 7.0) and catalase treatment. These treatments abolished the antibacterial activity, suggesting that organic acids contribute to the observed inhibitory effects.

The combined analysis of growth curves, pH profiles, and well diffusion antibacterial tests demonstrated a positive correlation between incubation time (16 to 48 h) and the diameter of inhibition zones against PA and SA. This indicates a time‐dependent accumulation of antibacterial metabolites. Notably, strains showing significant pH reductions (LRM, LR, LCP, and LPC) also displayed potent antibacterial activity, supporting the hypothesis that organic acids are the primary inhibitory compounds. Conversely, LA and BB, which exhibited no detectable antibacterial activity, displayed distinct growth and pH patterns. Specifically, LA maintained a relatively higher pH (4.0–4.5), suggesting lower organic acid production, while BB showed a unique growth profile and pH trajectory, potentially indicative of alternative metabolite production or the absence of antibacterial compounds. These findings provide compelling evidence for optimizing the extraction of antibacterial metabolites by harvesting during the late stationary phase.

### 3.4 MIC and MBC of Lyophilized Probiotic CFSs

Based on the findings presented in Figure 1c, LRM, LR, LCP, and LPC exhibited both inhibitory and bactericidal capacities against gram‐positive (SA and MRSA) and gram‐negative (PA and PAO1) pathogens. Only BB did not show any detectable antibacterial activity.

The inhibitory and bactericidal activities of the majority of probiotic strains were largely unaffected by the antibiotic resistance status of the target bacteria. LPC, LCP, and LRM strains had stronger pathogen inhibitory and bactericidal activity compared to LR and LA. Notably, LPC inhibited and killed all four tested pathogens (PA, SA, MRSA, and PAO1) at the same concentrations: MIC = 6.25 mg/mL and MBC = 12.5 mg/mL, respectively. LRM performed a similar activity to LPC, except against SA, for which the MBC was 100 mg/mL (Table 3).

**Table 3:** MIC and MBC values of lyophilized CFSs against pathogenic strains.

Pathogenic strains	Probiotic strains									
	LPC		LCP		LRM		LA		LR	
	MIC	MBC	MIC	MBC	MIC	MBC	MIC	MBC	MIC	MBC
*P. aeruginosa* ATCC27853	6.25	12.5	6.25	12.5	6.25	12.5	12.5	25	12.5	25
*P. aeruginosa* PAO1	6.25	12.5	6.25	25	6.25	12.5	—	12.5	—	12.5
*S. aureus* ATCC25923	6.25	12.5	12.5	25	6.25	100	12.5	25	12.5	50
MRSA	6.25	12.5	6.25	25	6.25	12.5	12.5	25	25	50

Abbreviations: MBC (mg/mL), minimum bactericidal concentration; MIC (mg/mL), minimum inhibitory. concentration;

Statistical analysis was performed to compare the MICs and MBCs of the different CFSs against each pathogen. No significant difference was observed in the bactericidal effect of CFSs against the tested pathogens (*p* = 0.1739). However, a significant difference was found in the MICs of the CFSs against the tested pathogens (*p* < 0.0001).

The antibacterial activity of serially diluted, neutralized (pH 7.0), and catalase‐treated lyophilized CFSs was abolished against all tested pathogens.

A significant finding of these results is that LRM, LR, LCP, and LPC exhibited antibacterial efficacy against both gram‐positive and gram‐negative bacteria, demonstrating the broad‐spectrum potential of these postbiotics for therapeutic applications. Notably, while drug‐resistant strains often exhibit recalcitrance to last‐line antibiotics, these CFSs displayed both inhibitory and bactericidal activity at comparable concentrations across different bacterial groups. Specifically, the antibacterial activity of these strains, particularly LPC, remained largely unaffected by the antibiotic resistance status of the target pathogens, including MRSA and PAO1. Furthermore, LPC inhibited and killed these pathogens at equal MIC and MBC values, emphasizing its strong and consistent antibacterial potential. The low and consistent MIC and MBC values observed for LPC highlight its promise as a candidate for novel antimicrobial development.

Consistent with previous findings, neutralization and catalase treatment abolished the antibacterial activity of the lyophilized CFSs, confirming the critical role of organic acids as the primary inhibitory compounds. This also suggests that any additional antibacterial metabolites produced are likely unstable at neutral pH or susceptible to catalase degradation.

An important observation was the enhanced antibacterial activity of the lyophilized CFSs in the microdilution assay compared to the supernatant used in the well‐diffusion test. This suggests that lyophilization concentrates the active components, thereby significantly amplifying their antibacterial potency. For instance, LA, which exhibited no activity in the well‐diffusion assay (Figure 1c), demonstrated antibacterial activity in the microdilution assay (Table 3). Given that lyophilization is a widely used method for preserving biological materials, this finding indicates that the antibacterial compounds remain stable and are likely concentrated during this process. However, it is important to acknowledge that differences in assay methodologies may have also contributed to the observed variations in antibacterial activity.

### 3.5 Durability of the Inhibitory Effect of Lyophilized CFSs

**Figure 2:**
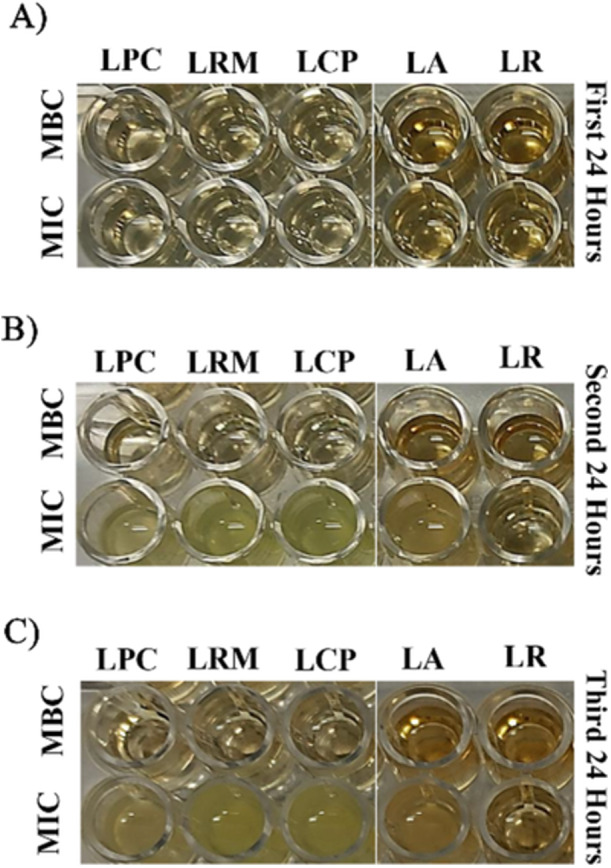
Durability of the antibacterial activity of lyophilized CFSs against *Pseudomonas aeruginosa* ATCC 27853 over 72 h (A–C). Wells at the minimum inhibitory concentration (MIC) exhibited turbidity after 48 h, indicating bacterial regrowth (B), Wells at the minimum bactericidal concentration (MBC) remained clear throughout the 72‐h incubation period, demonstrating sustained bactericidal activity (C).

The durability of the inhibitory effect of the lyophilized CFSs against PA was evaluated over 72 h. As anticipated, wells containing MBC from the CFSs remained clear throughout the 72‐h incubation, confirming sustained bactericidal activity. Conversely, wells containing MIC of the CFSs remained free of turbidity only during the initial 24 h. Subsequently, visible turbidity developed in these wells during the 24–48 and 48–72 h intervals, indicating bacterial regrowth and metabolic activity of PA (Figure 2). Concomitantly, the pH of the MIC wells increased to a range of 8.0–9.0, whereas the pH of the MBC wells remained relatively stable, ranging from 3.0 to 5.0.

This assay effectively distinguished the bactericidal (MBC) and inhibitory (MIC) actions of CFSs, highlighting the effects of increasing concentrations of antibacterial metabolites, primarily organic acids, as well as the time‐dependent nature of MIC. While MBC reflects a sustained killing effect, MIC provides only temporary inhibition. The stable pH observed in MBC wells indicates a lack of bacterial metabolic activity and underscores the significant role of organic acids in bactericidal action.

### 3.6 Chemical Analysis of Lyophilized CFSs

The chemical composition of the most effective supernatants (LCP, LPC, and LRM) was analyzed using gas chromatography‐mass spectrometry (GC‐MS). Several organic acids, including lactic acid, acetic acid, stearic acid, and palmitic acid, as well as various alcohol derivatives, were detected in varying relative abundance. 2‐Pyrrolidonone was also identified in all three CFSs. The relative abundance of each identified compound in LCP, LPC, and LRM is presented in Table 4.

**Table 4:** Chemical composition of LCP, LPC, and LRM supernatants.

No.	Compound	Probiotic strains Peak area (%)			
		RT (min)	LRM	LPC	LCP
1	Acetic acid	2.009	19.22	3.90	7.34
2	3‐Methyl‐2‐hexanol	6.92	8.63		
3	2‐IsoPropyl alcohol	7.505			34.22
4	Octadecanoic acid (Stearic acid)	8.566		46.45	
5	Lactic acid	9.021	30.47		17.09
6	1‐Methoxy‐2‐methylpropane	9.132	12.85		
7	2‐Butanol	9.761			24.40
8	2‐Pyrrolidonone	11.09	3.52	7.61	5.17
9	3‐Methyl‐3‐oxetane methanol	11.306			3.14
10	2,3‐Dihydro‐2,5‐dihydroxy‐6‐methyl‐4 pyran‐4‐	12.215	1.53		
11	Hexadecanoic acid (Palmitic acid)	23.616		33.24	
12	Octanamine (1‐amino octane)	23.627	14.55		

Abbreviations: LCP, *Lacticaseibacillus* casei LC03; LPC, *Lacticaseibacillus* paracasei BGP 1; LRM, *Lacticaseibacillus* rhamnosus LR 06.

By comparing the organic acids in these three strains, it was found that LRM supernatant had the highest percentage of acetic acid (19.22%) and lactic acid (30.47%). For LCP, acetic acid and lactic acid were detected at 7.34% and 17.09%, respectively. 34.22% isopropyl alcohol was also identified in this sample. The supernatant of LPC contained different types of organic acids. It was full of stearic acid (46.45%) and palmitic acid (33.24%) and lacked lactic acid, unlike LRM and LCP. The abundance of acetic acid was only 3.90% (Table 4).

Although compound 2‐Pyrrolidonone (also known as butyrolactam) was found in all strains, its amount was higher in LPC than in the others (7.61%) (Table 4). In summary, these results confirm the crucial role of organic acids in the antibacterial activity of CFSs, with lactic acid dominating in LRM, mixed lactic and acetic acids in LCP, and stearic acid in LPC, consistent with the results of neutralization and catalase assays.

The GC‐MS analysis of the supernatant accounts for the observed variation in antibacterial activity, reflecting differences in the types and concentrations of organic acids and other metabolites produced by each strain. The distinct results shown in Table 4 indicate that each strain possesses a unique metabolic profile. For instance, the abundance of stearic and palmitic acids and the absence of lactic acid in the LPC sample suggest a metabolic pathway distinct from that of LRM and LCP. The detection of multiple organic acids and alcohol derivatives implies potential synergistic antibacterial effects. Notably, the presence of 2‐pyrrolidone, particularly in high abundance in LPC, warrants further investigation, as it may contribute to antibacterial activity or exert other biological effects. Collectively, these results, along with neutralization and catalase assays, indicate that organic acids contribute substantially to the antibacterial activity of CFSs, with lactic acid predominating in LRM, mixed lactic and acetic acids in LCP, and stearic acid in LPC.

### 3.7 Protein Content Concentration of CFSs

**Figure 3:**
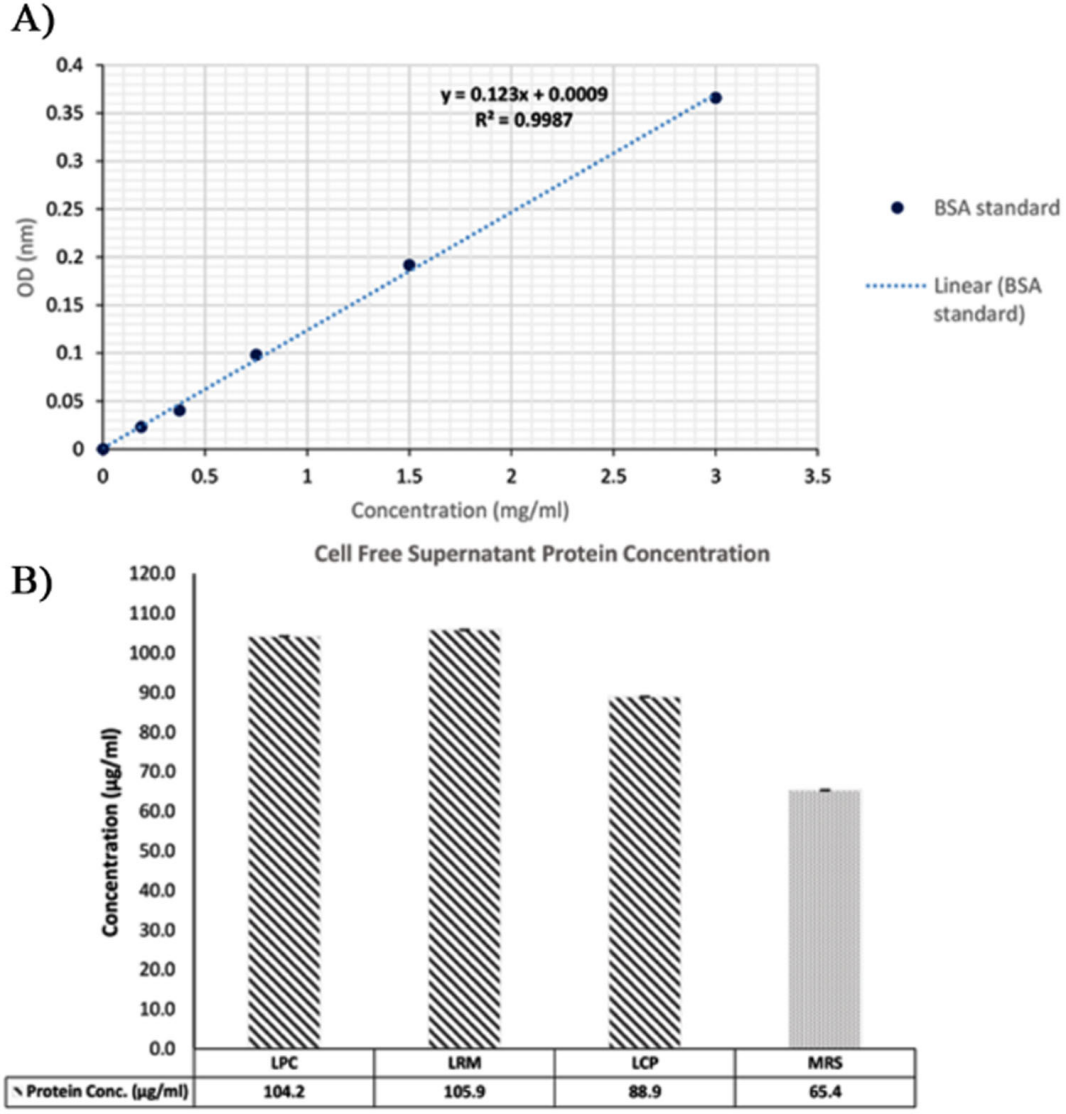
Protein concentration in probiotic cell‐free supernatants based on bovine serum albumin (BSA) standard curve. Standard Curve of Bovine Serum Albumin (BSA) Protein (A), Protein concentration in cell‐free supernatants of *Lacticaseibacillus paracasei* (LPC), *Lacticaseibacillus rhamnosus* (LRM), and *Lacticaseibacillus casei* (LCP) compared to MRS culture medium (B).

Figure 3 illustrates the BSA standard curve and the corresponding protein concentrations for each CFS sample. The protein concentration in each CFS sample (LCP, LPC, and LRM) was determined using a BSA standard curve, which ranged from 0 to 3000 µg/mL and had a high coefficient of determination (*r*² = 0.9987). Absorbance was measured at 595 nm. All CFS samples were found to contain proteinaceous components. The protein concentrations in LPC and LRM samples were similar (104.2 and 105.9 µg/mL, respectively) and higher than that of the MRS culture medium (65.4 µg/ml). The lowest concentration was detected in LCP (89 µg/mL). These results may reflect strain‐specific variations in bacterial protein production or extracellular secretion. Further research is needed to identify and characterize these proteins and their activity.

## 4 Discussion

Chronic skin wounds impose a substantial economic burden on healthcare systems, accounting for approximately 1%–3% of annual healthcare expenditures (Olsson et al. 2019). These costs are further amplified when wounds become infected with opportunistic bacterial pathogens. The emergence and spread of antibiotic‐resistant bacteria, exacerbated by the overuse and inappropriate prescription of antibiotics, significantly complicates the management of these infections. The resistance patterns observed in MRSA and PAO1 (Table 2) in this study underscore their serious clinical implications and point out the urgent need for alternative strategies to combat antibiotic resistance.

Although limited studies have been conducted in the last two decades to investigate the effect of probiotics and their derivatives on wound healing and infection control, these studies have yielded noteworthy results. A series of studies have demonstrated the efficacy of some probiotics in wound healing and even in the management of wound infections (Oryan et al. 2019; Sen 2019; Algburi et al. 2021). *Lactiplantibacillus plantarum*, LA, LRM, LR, LCP, and LPC are among the most common species used in these studies (Roager et al. 2014; Lukic et al. 2017; Fijan et al. 2019; Oryan et al. 2019; Sen 2019; Barzegari et al. 2020; Algburi et al. 2024). Nevertheless, only a limited number of probiotic strains available in the market have been recognized as effective for managing specific diseases. Furthermore, each strain possesses unique characteristics and produces various bioactive compounds in differing quantities. These factors may influence its effectiveness and efficiency in controlling infections and promoting the healing of chronic wounds.

In the present study, the potential of common probiotic strains available in the Iranian food and drug market (LR LRE 02, LA LA3, LCP LC03, LP BGP 1, LR LR 06, and BA BB12) was evaluated to produce antibacterial metabolites against key skin wound pathogens (SA, PA, MRSA, and PAO1). The optimal production time of these metabolites, together with their bactericidal and inhibitory concentrations in the supernatant, was investigated. Additionally, the stability of the inhibitory effects of these probiotic supernatants, referred to as postbiotics, was assessed.

The probiotic's growth curve, pH profile, and agar well diffusion assay demonstrated a correlation between the logarithmic growth phase, decreasing pH, and increasing diameter of pathogen growth inhibition zones. This trend continued into the stationary phase. These observations suggested that acidic metabolites were likely responsible for the observed antibacterial properties of the postbiotics. This hypothesis was supported by the absence of pathogen growth inhibition observed after the supernatant neutralization and catalase treatment. The quantity of these antibacterial metabolites produced during fermentation was highest in the stationary phase. Therefore, the 48th hour of growth was selected as the optimal time for supernatant extraction. These findings are consistent with previous research. Tejero‐Sariñena et al. (2012) reported that the antagonistic effect of the tested probiotics was associated with CFS pH reduction and organic acid production following glucose fermentation, observing a reduced antagonistic effect after CFS neutralization. Similarly, Hor and Liong demonstrated that lactic acid and acetic acid, produced by lactic acid bacteria, inhibit the growth of the dermatological pathogen SA by lowering the environmental pH, also noting a decreased inhibitory effect after CFS neutralization. However, unlike our findings, they also detected hydrogen peroxide and diacetyl in neutralized CFS (Hor and Liong 2014). Taken together, these findings propose that organic acids are the predominant antibacterial metabolites in the present study, although other compounds such as hydrogen peroxide, diacetyl, and bacteriocins have been reported to have antibacterial activity under different conditions.

To characterize the antibacterial acidic metabolites produced, the supernatant content of three superior strains (*L. casei* LC03, *L. paracasei* BGP 1, and *L. rhamnosus* LR 06) was chemically analyzed. This analysis revealed a high abundance of various organic acids, including acetic acid, lactic acid, stearic acid, and palmitic acid, along with lower concentrations of alcoholic compounds and 2‐Pyrrolidonone (Table 4). The supernatant of *L. rhamnosus* LR 06 (LRM) showed the highest relative abundance of both acetic acid and lactic acid. In the *L. casei* LC03 (LCP) supernatant, acetic acid and lactic acid were also detected, along with isopropyl alcohol. Although isopropyl alcohol is a known antibacterial compound (Qadi et al. 2023), its presence did not correlate with observed antibacterial activity following supernatant neutralization. Similarly, neutralization experiments indicated that other components, such as octanamine and 2‐Pyrrolidonone, detected in LRM, did not contribute to the observed antibacterial activity. Alternatively, their effect may be influenced by acidic conditions. Unlike LRM and LCP, the *L. paracasei* BGP 1 (LPC) supernatant contained stearic acid, palmitic acid, and acetic acid but lacked lactic acid. It is important to note that the protein content found in the supernatants revealed no effects under neutral conditions. Previous studies have established the antimicrobial properties of lactic acid and acetic acid (Hor and Liong 2014; Lopes 2017; Shokri et al. 2018; Qadi et al. 2023; Rahman et al. 2025), supporting our findings. For example, Lopes et al. (Lopes 2017) investigated the effect of probiotic supernatants on skin pathogens, including PA and MRSA. They attributed the observed antibacterial activity to the production of lactic acid and acetic acid. However, they reported *L. paracasei* L‐26 as a particularly prolific producer of both lactic acid (157.6 mmol/L) and acetic acid (57.45 mmol/L), a finding that differs from our quantitative results.

While organic acids' precise mechanism of action remains incompletely understood, existing research indicates that acidification of the pathogen's microenvironment, regardless of cell wall structure, creates conditions detrimental to growth and activity (Bajpai 2016). Organic carboxylic acids exhibit antimicrobial potential due to the lipophilic nature of their undissociated form (RCOOH), which facilitates passive diffusion across the microbial plasma membrane. Consequently, carboxylic acids are generally considered more effective antimicrobial agents than strong mineral acids, as the latter primarily exert their toxic effects on the external cell surface (Hirshfield et al. 2003; Traisaeng et al. 2019). Consistent with these established principles, the present research revealed that the inhibitory effect of the postbiotic on pathogen growth persisted for only 24 h. Subsequent increases in environmental pH beyond this timeframe correlated with a loss of inhibitory activity and the resumption of pathogen growth. This observation aligns with the finding that, following acid removal, damaged cells can fully recover their physiological functions (Hirshfield et al. 2003; Wesche et al. 2009). We hypothesize that this decline in efficacy may be attributed to a combination of factors, including a decrease in the concentration or activity of CFS organic acids, the emergence of more resistant pathogen subpopulations (changes in either gene expression or cell physiology), and/or the production of alkaline metabolites by the pathogens. This interpretation is further supported by the findings of Qadi et al. (Qadi et al. 2023), who demonstrated stable antibacterial activity of CFS within a pH range of 3–6, with a complete loss of inhibition at pH 9. Similarly, Traisaeng et al. (Traisaeng et al. 2019) reported that *Lactobacillus johnsonii* NCC 533 inhibited the growth of *Salmonella enterica* serovar Typhimurium SL1344 only at a low pH of 4.5 and not at pH 5.6.

Beyond their inhibitory activity, our probiotic supernatants demonstrated a significant bactericidal effect against all tested standard and resistant SA and PA skin wound pathogens. This bactericidal capability may be attributed to the nature and increasing concentration of organic acids within the supernatants. While the bactericidal effect is partly mediated by a reduction in environmental pH and its impact on the proton electrochemical gradient, it also results from direct effects on the bacterial cell membrane, including its phospholipids and lipopolysaccharides. Upon entering the cytoplasm, these acids induce acid stress, leading to significant changes in cytoplasmic osmolarity (osmotic stress), disruption of cellular homeostasis, inhibition of cytoplasmic enzyme activity, protein and DNA damage, free radical generation, and ultimately, cell death (Hirshfield et al. 2003; Boomsma 2015; Stanojević‐Nikolić et al. 2016; Traisaeng et al. 2019). Furthermore, these acids can increase membrane permeability, facilitating the internalization of other antimicrobial agents present in the supernatant, such as the identified alcohols and peptides (Neal‐McKinney et al. 2012; Lenzmeier et al. 2019). At sufficiently high concentrations, these compounds likely cause bacterial cell death. Consistent with this, Lenzmeier et al. (Lenzmeier et al. 2019) demonstrated that the production of sufficient lactic acid by *Lactobacillus gasseri* 63AM during 48 h of growth disrupted the *P. aeruginosa* PAO1 membrane, allowing the internalization of secondary inhibitory factors. This suggests that the activity of other compounds within the supernatant depends on the presence and action of the short‐chain organic acids produced during fermentation.

In our work, in addition to alcoholic compounds, 2‐Pyrrolidone was identified in the postbiotics of all three tested strains (LCP, LPC, and LRM). While this compound is a versatile heterocyclic amide with diverse pharmaceutical and industrial applications, its direct role as an antibacterial agent is not extensively documented. 2‐Pyrrolidone (butyrolactam) is structurally related to proline and has been identified as a metabolite of putrescine in mammalian systems. The pyrrolidone core is a common structural motif in various natural products derived from fungi (Hosseinzadeh 2017; Liu et al. 2024) and bacteria (Sathiyanarayanan et al. 2014; Zhang et al. 2019). Some pyrrolidone derivatives, such as 2‐pyrrolidone‐5‐carboxylic acid, have been found in lactic acid bacteria, including *Pediococcus*, *Lactobacillus* (e.g., *Schleiferilactobacillus harbinensis*), and co‐cultures of *LP* and *Propionibacterium jensenii*. Although some pyrrolidone derivatives exhibit a broad spectrum of biological activities, including inhibitory effects against *Pseudomonas* (Mohsenipour and Hassanshahian 2016; Lombardi et al. 2019; Mora‐Villalobos 2020; Tegegne and Kebede 2022), the observed antibacterial activity of our probiotic supernatants is more likely attributable to the synergistic action of the identified organic acids. These organic acids are key metabolites, enhancing the supernatant's efficacy by increasing membrane permeability. This increased permeability facilitates the entry of other compounds, such as 2‐pyrrolidone, which may then contribute to the overall antibacterial effect by influencing membrane integrity or other cellular processes. Thus, the organic acids play a crucial role in enhancing the antibacterial activity of the supernatant. However, further investigation is required to elucidate the precise role of 2‐pyrrolidone in this activity.

Protein concentration analysis of the studied postbiotics revealed a range of proteinaceous compounds, quantified from 89 µg/mL in LCP to 105.9 µg/mL in *LP*. These compounds likely include enzymes and bioactive peptides, typically composed of 2–20 amino acid residues. Notably, bioactive peptides have been reported to exhibit antioxidant properties (Fideler et al. 2019). Given that bacterial protein production is predominantly intracellular, the protein concentration in the supernatant remains comparatively lower (Mora‐Villalobos 2020). Neutralization of the supernatants abolished antibacterial activity, suggesting that protein concentrations alone were insufficient to elicit inhibition. Or perhaps their effect depends on acidic conditions. For instance, in the presence of organic acids, known to disrupt bacterial membranes, these proteinaceous compounds may gain entry into pathogenic cells and exert intracellular effects, akin to other supernatant constituents. Therefore, further characterization, including identification and functional analysis, is necessary to elucidate the precise antibacterial mechanisms associated with these postbiotics.

A remarkable finding of this study was the potent antibacterial activity of the LPC postbiotic against both gram‐positive and gram‐negative pathogens, despite the low concentration of acetic acid and the absence of detectable lactic acid. The non‐detection of lactic acid is likely attributable to the extended fermentation time. During prolonged fermentation, heterofermentative bacteria can convert lactic acid into secondary metabolites such as acetic acid, ethanol, and propionic acid. Additionally, lactic acid bacteria (LAB) produce small amounts of acetyl‐CoA, which contributes to synthesizing acetate, acetylated compounds, and fatty acids (e.g., palmitic and stearic acids) under microaerophilic conditions. It is hypothesized that during the 48‐h preparation of the probiotic culture, the lactic acid produced was either consumed by the bacteria or converted into other metabolites (Muller 2008; Krivoruchko et al. 2014). Notably, LPC exhibited potent antibacterial activity, inhibiting and killing all four tested pathogens (*PA*, *SA*, MRSA, and *PA* (PAO1)), at the same concentrations: MIC = 6.25 mg/ml and MBC = 12.5 mg/ml, respectively. These inhibitory and bactericidal concentrations were comparable to those observed for supernatants containing both lactic and acetic acid. This suggests that the antibacterial activity of the LPC postbiotic is mediated by mechanisms independent of high concentrations of these organic acids (lactic acid and acetic acid) and is not influenced by the antibiotic resistance profiles of the tested pathogens. This observation supports the hypothesis that the antimicrobial mechanism of the LPC supernatant, likely involving palmitic and stearic acids as primary active components, differs from that of conventional antibiotics, exhibiting a broader, less specific mode of action.

Palmitic and stearic acids have been previously detected in the culture media of several LAB strains (Rahman et al. 2025), including *Lactobacillus helveticus* (Sharma et al. 2014; Bajpai 2016), *Lactiplantibacillus plantarum* (Ramos et al. 2015; Poornachandra Rao et al. 2019), LCP (Hong‐Xin et al. 2015), LPC subsp. *paracasei* NTU 101 (Chang and Pan 2019), and *LP* MI29 (Kim et al. 2023). In addition, *LP* BGP1 has been shown to increase the abundance of palmitic and stearic acids in fermented beverages (Ziarno et al. 2020), suggesting that this species can produce these fatty acids in significant quantities.

Antimicrobial (Sheela et al. 2012; Chaudhary et al. 2020; Rahman et al. 2025; Tenea et al. 2025), antifungal (Raissa et al. 2020; Shah et al. 2024), and antiviral (Kim et al. 2023) properties of palmitic acid and stearic acid were indicated in a few previous studies (Chaudhary et al. 2020). For example, the antifungal effect of *L. plantarum* MYS44 CFS on *Aspergillus parasiticus* MTCC 411 referred to 15 bioactive components, including palmitic acid (Poornachandra Rao et al. 2019). Palmitic acid was also detected in *L. plantarum* 162 as a metabolite that helps control *S. typhimurium*, *E. coli*, and *S. sonnei* (Kanjan and Hongpattarakere 2016; Gonzalez 2020). *L. paracasei* MI29 isolated from feces helped to prevent influenza virus infection through palmitic acid (Kim et al. 2023). Although palmitic acid and LAB‐derived acid have been extracted from many biological sources, especially plants and bacteria, only a few have shown antibacterial effects. Furthermore, studies evaluating the antibacterial activity of these fatty acids have focused more on gram‐negative than gram‐positive pathogens. In this study, *LP* BGP 1 was identified as the main biological producer of these antibacterial metabolites. The effectiveness of this postbiotic against gram‐positive and gram‐negative bacteria, both standard and resistant strains, was revealed. This potential has not been reported previously for *L. paracasei* BGP 1.

In recent years, some researchers have suggested that probiotics, in addition to their direct antimicrobial effects on pathogens and their ability to modulate the host immune system, have the potential to repair the epithelial barrier through the activation of epithelial cells, stimulation of cell proliferation or migration, and subsequently enhancing collagen synthesis (Lukic et al. 2017). Moreover, studies conducted in animal model conditions have also demonstrated the effectiveness of a group of probiotics, including *Lactiplantibacillus plantarum*, in controlling infections, reducing inflammation, and promoting skin tissue regeneration in burn and diabetic wounds. These results have shown promising outcomes (Lopes 2017; Satish et al. 2017; Oryan et al. 2019; Ong et al. 2020).

Scientific evidence suggests that developing safe and innovative antibacterial treatments using probiotics or their products is feasible. Although clinical research has predominantly focused on live probiotic cells, mainly through oral applications, the use of postbiotics is gaining increasing attention. They have the potential to be regarded as a complement to or even an alternative to antibiotics. They offer reduced side effects and avoid the risk of inducing antibiotic resistance. Additionally, postbiotics may act as soothing agents, topical immunomodulators for damaged skin, and wound‐healing accelerators. Another point to consider is that the use of postbiotics in pharmaceutical and cosmetic skin products presents fewer challenges, including greater stability and reduced production costs, compared to formulations containing live cells. In this regard, it is essential to identify and screen promising probiotic strains and their derivatives for controlling pathogenic strains, establish effective dosing, and assess host cell and tissue toxicity, including interactions with cell lines exposed to wound pathogens. According to the findings of this study, LCP, LPC, and LRM all exhibited potent antibacterial activity, with LPC showing the most pronounced effect. LPC was unique in its ability to inhibit both gram‐positive and gram‐negative bacteria with no difference in efficacy across groups. Furthermore, LPC achieved this inhibition at a lower concentration compared to strains LRM and LCP. LPC BGP 1, a producer of potent antibacterial acidic metabolites, is a promising candidate for controlling skin wound pathogens through postbiotic‐based topical application. It warrants further investigation through formulation studies and clinical evaluation.

Infected skin wounds with bacterial pathogens are a costly challenge for the healthcare system. Unfortunately, many common antibiotics are no longer effective against some strains of the pathogen, especially multidrug‐resistant types. Therefore, it is necessary to find new and accessible alternatives. In addition to beneficial health effects, probiotics are rich sources of antibacterial metabolites. Referring to the present results, it suggests that bio‐based organic acids (lactic acid, acetic acid, palmitic acid, and stearic acid) have significant and nonspecific antibacterial activity. In particular, palmitic acid and stearic acid, produced by LPC BGP1, not only inhibit but also completely kill standard and resistant strains of PA and SA that infect skin wounds.

Generally, organic acids are safe, efficient, and readily available metabolites that probiotics generate easily to control infectious bacteria. The nonspecific function is a notable advantage that differentiates them from conventional antibiotics. If their potential is tested in vivo, they can be introduced as one of the most appropriate antibiotic complements or alternatives.

## 5 Conclusion

Unfortunately, many common antibiotics are no longer effective against certain pathogenic bacteria and have contributed to the emergence of antibiotic‐resistant strains. Therefore, it is essential to find new and effective alternatives. Postbiotics, compared with probiotics, are safer, more efficient, and associated with fewer side effects in controlling infectious bacteria, as they are rich sources of antibacterial metabolites. In this study, our results advocate that bio‐based organic acids have significant and nonspecific antibacterial activity. In particular, palmitic acid and stearic acid, identified in LPC BGP1 postbiotic, were primarily responsible for inhibiting and killing *PA* and *SA*, including antibiotic‐resistant types.

These findings highlight the capacity of *L. paracasei* BGP1 postbiotic as a promising nonliving option to conventional treatments for antibiotic‐resistant skin wound infections, especially in formulations of topical skin products, pending further clinical validation.

More broadly, probiotics and their naturally derived antimicrobials have the potential to be combined with conventional antibiotics and may represent a promising therapeutic strategy. Such combinations could enhance efficacy while reducing the required antibiotic dose, minimizing toxicity, side effects, and treatment costs.

## Author Contributions

**Farideh Mohammadhosseinzadeh:** methodology, software, supervision, project administration, writing – original draft, writing – review and editing, validation, data curation, formal analysis, conceptualization, investigation, visualization. **Ehsan Arefian:** project administration, writing – original draft, writing – review and editing, visualization, conceptualization, methodology, data curation, investigation, formal analysis. **Mouj Khaleghi:** project administration, writing – review and editing, writing – original draft, validation, methodology, investigation, data curation, visualization, formal analysis. **Hoda Keshmiri Neghab:** writing – original draft, writing – review and editing, visualization, validation, supervision, formal analysis, investigation. **Nasim Kashef:** conceptualization, methodology, supervision, writing – review and editing.

## Ethics Statement

The authors have nothing to report.

## Conflicts of Interest

The authors declare no conflicts of interest.

## English Language Editing

The authors used ChatGPT for language editing assistance to improve the grammar, clarity, and readability of the manuscript. All content was originally written by the authors, and they take full responsibility for the final version.

## Data Availability

All data generated or analyzed during this study are included in this published article.
